# Hepatitis B virus suppression through pEPI vectors and expression of small non-coding RNA molecules

**DOI:** 10.1186/2194-7791-1-S1-A8

**Published:** 2014-09-11

**Authors:** Kai Hensel, Andreas Klein, Lisa Willuhn, Susanna Prax, Andreas Jenke, Stefan Wirth, Jan Postberg

**Affiliations:** University Children’s Hospital Witten/Herdecke, Wuppertal University, Wuppertal, Germany

## Aims

Hepatitis B virus (HBV) infections in early childhood are associated with a high risk of chronification and the subsequent risk of cirrhosis and development of hepatocellular carcinoma (HCC) in adulthood. Immunization success rates are still suboptimal and treatment options bare considerable undesired side effects and drug resistance development. As a novel experimental therapeutic approach we investigated on the utilization of siRNAs in order to suppress HBV replication.

## Methods

In an experimental cell culture setting, we used the different expression vector systems for both transient and long-term expression of various small non-coding RNAs in order to suppress HBV replication in HBV-transgenic murine hepatocytes. Several HBV-specific shRNAs and microRNAs were designed, transfected in combination with different tissue-specific gene promotors and compared to nucleoside analogon therapy effects. Gene expression profiles for 84 genes relevant for HCC development were subsequently analyzed.

## Results

HBV virus replication was suppressed by all siRNAs expressed as indicated by diminished HBsAG titers in cell culture supernatant. Imitations of miR-122, miR-26a1 and miR-30-a containing HBV-specific attachment sequences were cloned in pENTRY vectors under U6 and H1 RNA polymerase III promotor control. H1 promotor expression of microRNAs was slightly superior to U6 promotor expression of microRNAs. shRNAs lead to stronger genetic dysregulation than HBV-specific microRNA imitations.

## Conclusion

siRNAs can sufficiently suppress HBV replication in HBV-transgenic murine hepatocytes. MicroRNAs lead to less undesired off-target effects than shRNAs and are a promising therapeutic agent for further experimental investigations.

